# Diffusion tensor imaging measures of white matter compared to myelin basic protein immunofluorescence in tissue cleared intact brains

**DOI:** 10.1016/j.dib.2016.12.018

**Published:** 2016-12-18

**Authors:** Eric H. Chang, Miklos Argyelan, Manisha Aggarwal, Toni-Shay S. Chandon, Katherine H. Karlsgodt, Susumu Mori, Anil K. Malhotra

**Affiliations:** a Center for Psychiatric Neuroscience, The Feinstein Institute for Medical Research, Northwell Health, 350 Community Drive, Manhasset, NY 11030, USA; b Division of Psychiatry Research, Zucker Hillside Hospital, Northwell Health, 75-59 263rd Street, Glen Oaks, NY 11004, USA; c Hofstra Northwell School of Medicine, Departments of Psychiatry and Molecular Medicine, Hofstra University, Hempstead, NY, USA; d Russell H. Morgan Department of Radiology and Radiological Science, Johns Hopkins University School of Medicine, Baltimore, MD 21205, USA; e F.M. Kirby Research Center for Functional Brain Imaging, Kennedy Krieger Institute, Baltimore, MD 21205, USA

**Keywords:** DTI, CLARITY, Multimodal imaging, Myelination, Radial diffusivity

## Abstract

We provide datasets from combined *ex vivo* diffusion tensor imaging (DTI) and Clear Lipid-exchanged, Anatomically Rigid, Imaging/immunostaining compatible, Tissue hYdrogel (CLARITY) performed on intact mouse brains. DTI-derived measures of fractional anisotropy (FA), radial diffusivity (RD), and axial diffusivity (AD) were compared to antibody-based labeling of myelin basic protein (MBP), as measured by fluorescence microscopy. We used a customized CLARITY hydrogel solution to facilitate whole brain tissue clearing and subsequent immunolabeling. We describe how CLARITY was made compatible with magnetic resonance imaging with the intention of facilitating future multimodal imaging studies that may combine noninvasive imaging with 3D immunohistochemistry. These data and methods are related to the accompanying research article entitled, ‘The role of myelination in measures of white matter integrity: Combination of diffusion tensor imaging and two-photon microscopy of CLARITY intact brains’ (E.H. Chang, M. Argyelan, M. Aggarwal, T-S. Chandon, K.H. Karlsgodt, S. Mori, A.K. Malhotra, 2016) [1].

**Specifications Table**

**Table:** 

Subject area	*Neuroscience, Radiology*
More specific subject area	*Diffusion tensor imaging and immunohistochemistry*
Type of data	*Table, fluorescence microscopy images, summary graphs*
How data was acquired	*Diffusion MRI was acquired on Bruker 11.7T NMR spectrometer. Two-photon laser microscopy images were acquired on Olympus FV-1000-MPE microscope.*
Data format	*Images and graphs*
Experimental factors	*Mice were transcardially perfused with a CLARITY liquid hydrogel, ex vivo brains were then immersed in PBS and 0.1 mM gadopentetate dimeglumine prior to DTI. During scanning, brains were immersed in Fomblin for susceptibility matching and to limit tissue dehydration.*
Experimental features	*TrackVis was used to create white matter regions-of-interest (ROIs) from DTI-derived FA maps. CLARITY images were analyzed using Imaris 8.0 with the Surfaces module to trace individual MBP-positive ROIs.*
Data source location	*Manhasset, New York, USA*
Data accessibility	*Data is provided in this article and is related to the research article* [1].

**Value of the data**

• This is the first dataset of CLARITY whole-brain MBP immunolabeling and the first study, to our knowledge, combining DTI with CLARITY in the same brain samples.
• CLARITY immunolabeling can be used in future studies to examine other protein targets found in brain structures with complex 3D architectures and distributions. This enables molecular analysis of brain targets across intact macroscopic circuits, while maintaining microscopic resolution.
• Our dataset demonstrates that within-subject multimodal imaging studies can provide useful insight into the biological basis of noninvasive imaging methods.

## 1 Data

**Fig. 1:**
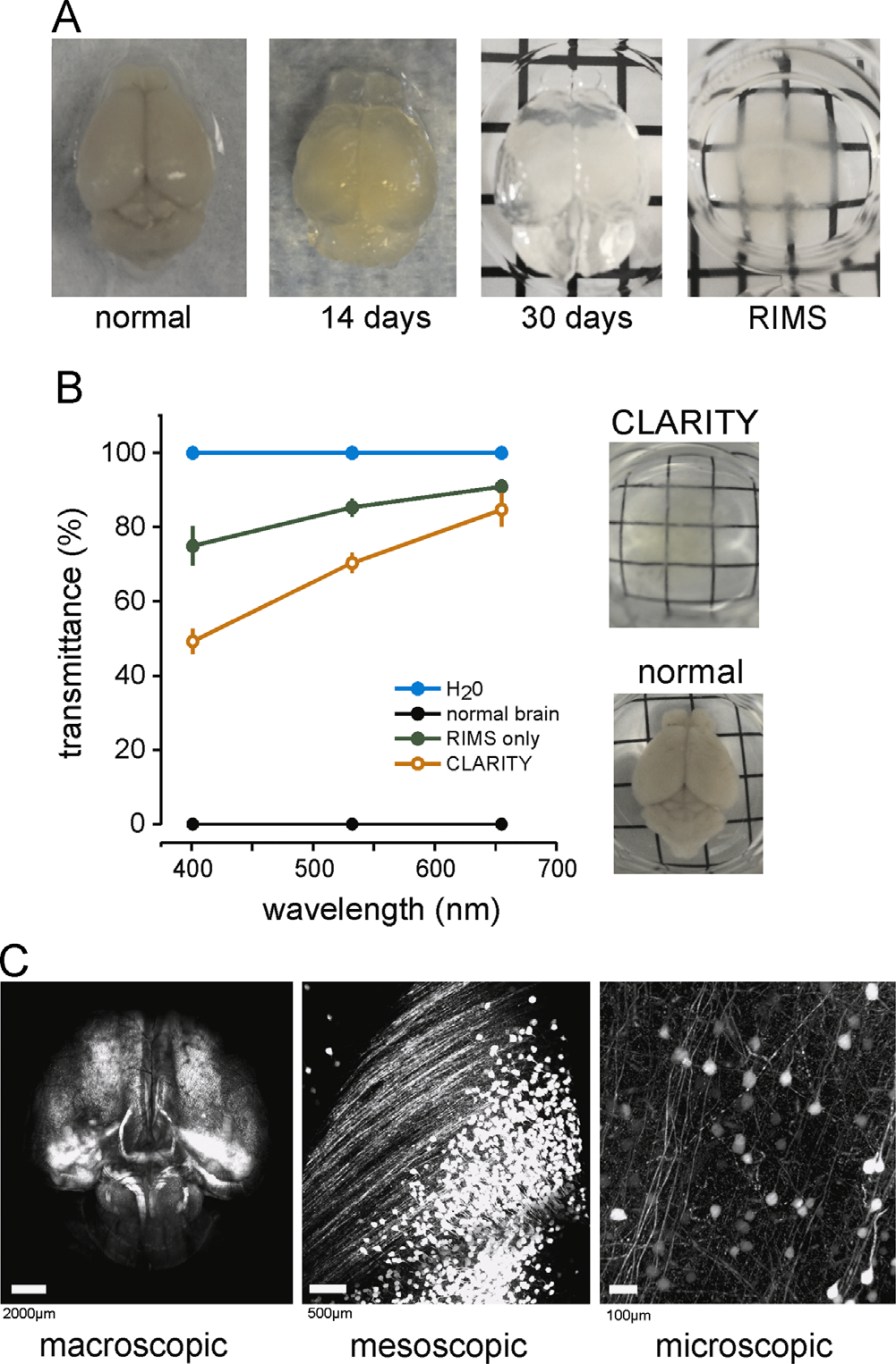
CLARITY clearing and Thy1-YFP-H images across multiple scales (A) Example images of normal PFA-fixed brain and CLARITY brains during the clearing process. (B) Plot shows mean±SEM tissue transparency of CLARITY samples at three laser wavelengths. Transmittance values were normalized to H_2_0. Images on the right show an example whole brain prior to clearing and a CLARITY-cleared brain. (C) Example images of a CLARITY Thy1-eYFP-H intact brain demonstrating the potential for examination of fluorescence on multiple biological scales.

**Fig. 2:**
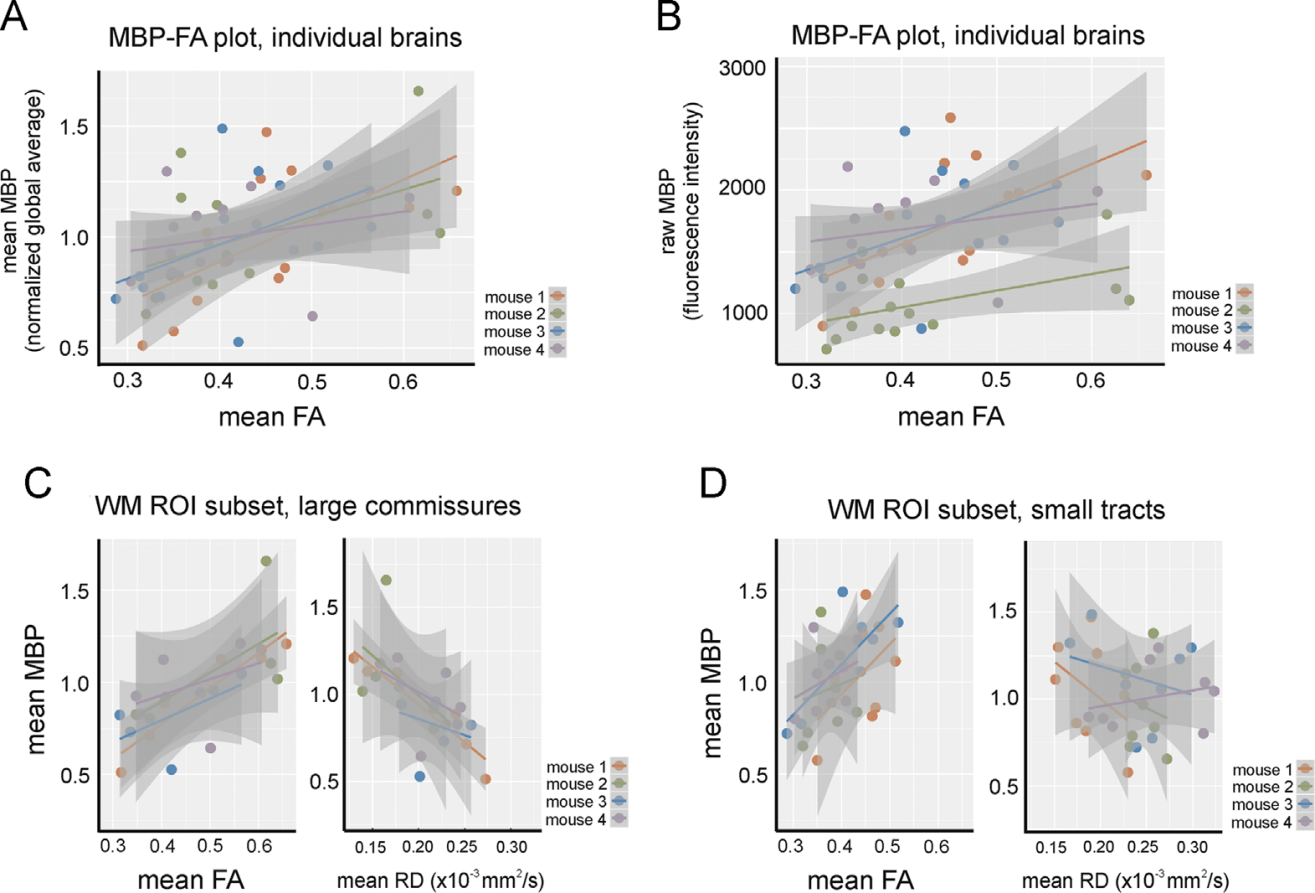
Raw MBP immunofluorescence and individual brain data from FA, AD, and RD correlations. (A) Scatter plots showing MBP-FA correlations for each individual mouse brain across the entire set of WM ROIs. MBP immunofluorescence was normalized to a global average. (B) Scatter plots showing MBP-FA correlations for each individual mouse brain, with raw MBP fluorescence intensity values. (C) Scatter plots of MBP-FA and MBP-RD correlations for a subset of WM tracts (corpus callosum, fimbria, and anterior commissure) showing data from each individual brain, with MBP normalization to a global average. (D) Scatter plots of MBP-FA and MBP-RD correlations for a different subset of WM tracts (fornix, stria medullaris, fasciculus retroflexus, and mammillothalamic tract) showing data from each individual brain, with MBP normalization to a global average. For all plots, linear regressions are shown with 95% confidence intervals (grey).

**Fig. 3:**
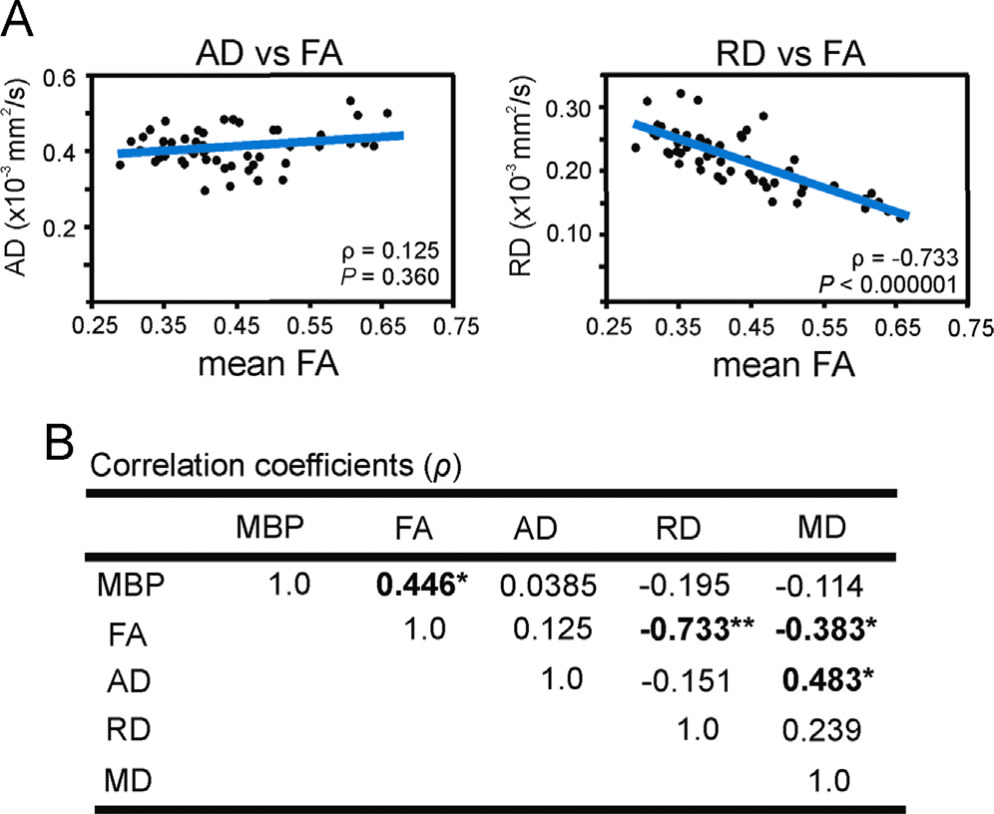
Additional FA and directional diffusivity correlation plots. (A) Correlation plots of directional diffusivity and FA measures. Left, FA did not correlate significantly with AD. Right, RD had a significant negative correlation with FA. (B) Correlation matrix for MBP, FA, AD, RD, and MD. Statistically significant correlations are indicated in bold, *P<0.005, **P<0.00001.

The dataset includes images, videos, and plots from experiments combining *ex vivo* DTI and two-photon microscopy of CLARITY mouse brains. Fig. 1, Fig. 2, Fig. 3 show both raw and analyzed data from these experiments indicating the relationship between various diffusivity measures and MBP immunofluorescence. Table 1 shows DTI-derived measures and MBP immunofluorescence values for major myelinated white matter tracts of the mouse brain.

Supplementary material related to this article can be found online at doi:10.1016/j.dib.2016.12.018.

The following is Supplementary material related to this article Video 1, Video 2.

Video 1 **3D visualization of CLARITY MBP-labeled whole mouse brain**. Imaris animation shows a MBP immunolabeled whole brain. Major myelinated white matter structures such as the hippocampal commissure, corpus callosum, and stria medullaris are plainly visible.

Video 2 **Optical section through CLARITY MBP-labeled mouse brain**. 1500 µm thick sagittal optical section near the midline showing many of the major myelinated white matter tracts used for comparison in this study. White matter in the cerebellum and brainstem (not quantified in this study) were also visibly labeled by MBP antibody..

## 2 Experimental design, materials and methods

### 2.1 Experimental design and animal subjects

For all experiments, we used C57BL/6J mice from Jackson Laboratories (Bar Harbor, ME). All animal procedures were approved by the Feinstein Institute Medical Research Institutional Animal Care and Use Committee and maintained according to National Institutes of Health guidelines. We first piloted our combined DTI and CLARITY [2] approach using *Thy1*-eYFP-H mice (*n*=3) that endogenously produce fluorescence signal without immunolabeling (Fig. 1**C**). Once we finalized a working protocol that enabled DTI followed by CLARITY, we used four C57BL/6J mice for the main experiments. The mice were first perfused with a CLARITY liquid hydrogel solution, then brains were removed and underwent *ex vivo* DTI scanning. Subsequently the CLARITY hydrogels were polymerized, tissue cleared, MBP immunolabeled, and finally imaged intact using a two-photon laser scanning microscope. Details of each step can be found below and also in the research article [1].

### 2.2 ex vivo DTI

Mouse brains were scanned in a CLARITY liquid hydrogel containing 4% paraformaldehyde and 0.1 mM gadopentetate dimeglumine (Gd-DTPA). The Gd-DTPA was used as a T1 shortening contrast agent to achieve shorter repetition times (TR), thereby allowed faster DTI acquisitions while maintaining good signal-to-noise [3]. Diffusion-weighted magnetic resonance (MR) images were acquired on an 11.7 T NMR spectrometer (Bruker BioSpin, Billerica, MA) using a three-dimensional gradient-and-spin-echo (3D DW-GRASE) sequence with twin navigator-echo phase correction [3], along 15 independent directions. A b-value of 1500 s/mm^2^ was used and scanning time was ~16.5 h for each mouse brain.

### 2.3 CLARITY clearing and MBP immunolabeling

CLARITY tissue clearing and immunolabeling were performed as previously described [2] with important modifications made to the hydrogel solution in order to obtain sufficient whole brain immunolabeling, while also maintaining tissue rigidity. We experimented with different concentrations of paraformaldehyde (PFA) and bis-acrylamide before arriving at an optimal hydrogel mixture containing 4% paraformaldehyde, 1.75% acrylamide, 0.01875% bis-acrylamide, 0.25% VA-044 initiator (Wako Chemicals USA) and 1X PBS. This resulted in a hydrogel-tissue matrix that facilitated lipid clearance and allowed for deep immunolabeling of whole mouse brains. Instead of electrophoretic tissue clearing (ETC) at high temperature, we used passive clearing at 37 °C (without agitation) in order to minimize changes in overall brain volume that commonly occur with ETC. While this passive technique is markedly slower, we found that it achieved excellent tissue transparency and structural preservation without tissue inflation (Fig. 1**A** and **B**). Following clearing, intact brains were immunlabeled with anti-MBP (1:50, EMD Millipore), followed by secondary antibody labeling with AlexaFluor 633 (1:50, Life technologies). The entire clearing and immunolabeling procedure took approximately 80 days to complete for each whole brain. Once MBP labeled, brains were immersed in refractive index matching solution (RIMS; [4]) and imaged using an Olympus FV1000-MPE with Mai Tai DeepSee Ti:Sapphire laser (SpectraPhysics).

### 2.4 DTI-CLARITY analysis

From the diffusion-weighted MR images, maps of FA, AD, RD, and MD (Table 1; Fig. 3) were calculated using FSL (http://www.fmrib.ox.ac.uk/fsl) software. TrackVis (http://www.trackvis.org) was used for post hoc calculations and visualization. White matter regions-of-interest (ROIs) were drawn in TrackVis from the FA map. For visualization and analysis of two-photon image stacks, we used Imaris 8.0 software (Bitplane). ROI-based analyses (Fig. 2) were conducted in the DTI space and CLARITY space with statistical comparisons using Spearman׳s rank correlations or mixed effect models performed in R software (version 3.0). Statistical significance for all tests was defined as *P*<0.05.

## Figures and Tables

**Table 1:** **Mean values of DTI-derived metrics and MBP immunofluorescence** Table displaying mean±SEM values of DTI measures and mean MBP immunofluorescence for each WM ROI identified in this dataset. Mean values are averaged from the four mouse brains analyzed in this study. AD, RD, and MD values are in units of ×10^−3^ mm^2^/s. Raw MBP fluorescence intensities are unit-less with a range of 0–4095.

**White matter ROI**	**FA**	**AD**	**RD**	**MD**	**MBP**
Corpus collosum, genu	0.41±0.0042	0.39±0.0086	0.22±0.0085	0.28±0.0081	1333±239
Corpus collosum, body	0.34±0.012	0.39±0.010	0.24±0.012	0.29±0.011	1139±162
Corpus collosum, splenium	0.35±0.013	0.41±0.0087	0.25±0.0060	0.30±0.0061	1231±115
Anterior commissure, posterior	0.53±0.030	0.44±0.025	0.18±0.0079	0.27±0.0090	1609±193
Fimbria, left	0.56±0.024	0.46±0.027	0.17±0.016	0.29±0.0031	1707±196
Fimbria, right	0.58±0.025	0.45±0.019	0.15±0.011	0.27±0.027	1739±225
Fornix, left	0.40±0.025	0.41±0.016	0.21±0.019	0.27±0.015	1738±367
Fornix, right	0.46±0.020	0.40±0.031	0.21±0.025	0.24±0.015	1674±295
Stria medullaris, left	0.41±0.23	0.43±0.024	0.24±0.025	0.29±0.020	1935±266
Stria medullaris, right	0.41±0.026	0.43±0.028	0.23±0.019	0.28±0.018	1734±236
Fasciculus retroflexus, left	0.42±0.026	0.36±0.027	0.21±0.025	0.27±0.032	1709±214
Fasciculus retroflexus, right	0.42±0.031	0.35±0.022	0.23±0.033	0.25±0.021	1664±206
Mammillothalamic tract, left	0.33±0.015	0.42±0.026	0.27±0.021	0.32±0.023	1172±222
Mammillothalamic tract, right	0.33±0.018	0.42±0.014	0.26±0.020	0.32±0.014	1306±205

## Footnotes

Transparency document Transparency data associated with this article can be found in the online version at doi:10.1016/j.dib.2016.12.018.
