# Supplementary material for: Diffusion tensor imaging measures of white matter compared to myelin basic protein immunofluorescence in tissue cleared intact brains
Source: Data Brief. 2016 Dec 18;10:438–43. doi: 10.1016/j.dib.2016.12.018 (PMC5198630; doi:10.1016/j.dib.2016.12.018)
Supplement: Supplementary file 3 — Supplementary material. [file mmc1.docx]

**Manuscript title:** ‘Diffusion tensor imaging measures of white matter compared to myelin basic protein immunofluorescence in tissue cleared intact brains’

**Conflicts of interest:** none
